# Inflammatory Bowel Disease On-Line Web-Based Guide to Health Professionals and Patients in Developing and African Nations

**Published:** 2020-03-11

**Authors:** AM Herman, AT Hawkins, SD James, BR Ballard, AE M’Koma

**Affiliations:** 1 Department of General Surgery, Kilimanjaro Christian Medical Center (KCMC), Moshi, Kilimanjaro, Tanzania; 2 Division of General Surgery, Section of Colon and Rectal Surgery, Vanderbilt University School of Medicine, Nashville, Tennessee, United States; 3 Department of Pathology, Meharry Medical College School of Medicine, Nashville General Hospital, Nashville, Tennessee, United States; 4 Department of Pathology, Microbiology, and Immunology, Tennessee Valley Health Systems VA, Medical Center, Vanderbilt University Medical Center, Nashville, Tennessee, United States; 5 Department of Pathology, Meharry Medical College School of Medicine, Nashville General Hospital, Nashville, Tennessee, United States; 6 Department of Surgery and Surgical Sciences, Meharry Medical College School of Medicine, Nashville, Tennessee, United States; 7 The American Society of Colon and Rectal Surgeons (ASCRS), Arlington Heights, IL 60005, United States; 8 The American Gastroenterological Association (AGA), Bethesda, MD 20814, United States; 9 The International Society of Colon and Rectal Surgeons (ISUCRS), 109 Partin Street, Chapel Hill, NC 27514, United States

**Keywords:** Inflammatory bowel disease, Ulcerative colitis, Crohn’s disease, Curriculum, E-web-based learning, E-learning technology, Developing and african nations, Environmental factors, Urbanization, Continued medical education

## Abstract

**Introduction:**

Inflammatory Bowel Disease (IBD) is recklessly evolving worldwide as incautious disaster, especially in developing nations as a regional duplicitous emergence disease. It has come to light that adaptive Western culture, rapid urbanization lifestyle in the developing nations has been seen to be associated with this increasing trend incidence. Apparent unclassified strategic challenge assessment of how key trends and uncertainties might lead the world over the next decades to help developing nations and plan for the long term. Healthcare professionals are faced with limited resource and unequipped laboratories for IBD diagnostics, prognostics and monitoring management. Limited knowledge on IBD among developing nation’s physician’s/healthcare providers is painstaking and indisputable challenge. With the emergence of advanced communications technology, the internet offers diverse, substantial, easily accessible, and educational resources that are more time- and cost-efficient than conventional modes of knowledge acquisition. An On-Line Web-Based Resources about IBD, as a guide would greatly assist health professionals and patients.

**Methods:**

We performed a literature search according to PRISMA-P (preferred reporting items for review and meta-analysis and searches in PubMed (MEDLINE database) to identify and select peer-reviewed articles allied to web-based educational accoutrements for IBD.

**Results:**

In developing nations, locally trained physicians have limited knowledge on IBD. Mostly, IBD is not included in their training Core Curriculum and research in this field/area is limited in these countries. The healthcare approaches, both at the primary care and referral levels, many times lack the essential regular clinical guidance and laboratory evaluation assessments needs for monitoring patients. Moreover, increasing treatment costs impose additional burden on the healthcare systems. Expensive pharmacological biosimilar and biologic agents/drugs, new treatment targets, and new quality indicators in patient health quality of life and care are significant challenge in addition to early manifestations of IBD are likely to be missed at most health institutions.

**Conclusion:**

We herewith summarize an on-line web-based e-learning guide for IBD-related educational resources to assist physicians, healthcare personnel and patients worldwide, especially in the developing nations where the epidemiological monitoring studies are limited, due to a lack of medical surveillance systems and reliable and unified registries and databases.

## 3. Background

Inflammatory Bowel Disease (IBD), encompassing Crohn’s Disease (CD) and Ulcerative Colitis (UC), has been reported expeditiously evolving over the last years gone by with the emergence of scientific and medical advances and challenges [1]. Additionally, the incidence and prevalence of IBD are sloping up throughout the world, especially in developing nations [2]. Until recently, incidence and prevalence of IBD was seen to be lower among developing nations [1, 2, 3, 4] compared to that in Western Europe and North America [5, 6]. Due to limited knowledge on IBD among developing nation’s physicians, case reporting in these nations has been sporadic, and IBD has unknowingly been dealt and treated as a bacterial or parasitic infection [7–27]. Inadvertently, this contributes to severe delay in diagnosis and treatment [7, 8, 27, 28]. It is now known that IBD incidence in developing nations is higher than that reported and is rapidly rising [4, 7, 8]. Clearly unprecedented, apparently there is an egressing increase in the incidence and prevalence of IBD across Africa [3, 8–26], which includes CD and UC [1, 2].

From 1975–1980, Wright et al. studied IBD incidence in the Gastrointestinal (GI) Clinic of Groote Schuur Hospital, Cape Town, South Africa (SA) [19, 29]. They reported the incidence of CD and UC to be 117 and 220 cases, respectively. Among these patients, 72% and 60% were Caucasians, 37% and 37% were multiracial (mixed-race), and 1% and 3% were black, for each condition. The incidence for the multiracial and Caucasian population groups was reported to be 0.4 and 0.9 for CD, and 1.3 and 2.4 for UC per 100,000 person-years during 1970–1974. During 1975– 1980, the incidence was 1.3 and 1.2 for CD for each group, and 1.6 and 2.1 for UC for each group per 100,000 person-years. These differences with time in IBD incidence (1970–1974 to 1975–1980) in SA were significant (p< 0.05). It is unclear whether this increase was due to changing disease pattern or the increase in awareness and/or availability of testing. However, despite an increased IBD awareness, there is a significant delay in diagnosis, resulting in insufficient/ inadequate data to calculate basic statistics about IBD incidence for e.g. the entire African population [3, 8–26].

In recent years, multiple new therapies regimes have been appropriated for IBD, new therapeutic targets have been approved with the “treat-to-target” concept, and drug monitoring has been put into effect in IBD treatment [30, 31]. Likewise, IBD quality indicators aiming to advance and improve patient care have been refined [32, 33]. With the emergence of advanced communications technology, the Internet offers diverse, substantial, easily accessible, and accredited educational resources that are more time- and cost-efficient than conventional modes of knowledge acquisition.

Inflammatory bowel disease management is becoming increasingly complex, challenging, and importunate requiring complimentary education for practicing healthcare providers, especially gastroenterologists, both in the ambulatory care and hospital settings. Ellaway and Masters noted in their seminal review of e-learning medical education [34] that conventional Continuing Medical Education (CME) on topics related to IBD can be formal or informal. Formal education may involve face-to-face courses, conferences, seminars, workshops and grand rounds, while informal education may involve the reading of Journals and texts. Further, they also commented that obstacles to formal CME include increased professional workload, inability to get locum coverage, family commitments, and travel distance to conferences, and cost of attending courses [34]. Informal CME involves barriers to those of formal CME, but also include inadequate time, isolation and lack of access to professional colleagues, limited library resources, slow delivery of documents, shortage and/or lack of access to compatible technology, information technology scarcities, and cost.

It is necessary to enhance the knowledge of gastroenterology internists and residents in IBD treatment by providing them with extraneous discipline and increasing their liability to IBD patients during their core training. Furthermore, refined IBD fellowships in high-volume academic institutions offer opportunities for extensive training to those interested in careers focused on IBD. Other leverages for trainees include mentorship in IBD programs, electives in IBD, and courses with indepth track training in IBD like IBD Xcel [35–37].

Currently, there are online learning technologies that attempt to replicate and employ one-on-one interaction between trainees and instructors that normally occur during face-to-face classes [38]. Patients and physicians use the Internet to obtain information update and knowledge regarding various health contingencies. Unfortunately, to date, this approach is largely impractical in most developing countries and even in some parts of the developed countries such as United States, Canada and Europe. In a latest study of adult GI program directors and trainees in the United States, [39] pointed out that only one-third of the trainees were pleased with their level of liability to IBD cases and information, while more than half were unsatisfied managing conditions related to IBD (including the management of ileal pouch and stoma during staged restorative proctocolectomy, as well as pregnant and postoperative patients). On-line web-based resources were the first choice among the trainees as a primary information source for IBD clinical care [39]. Another recent study of 223 GI specialists in the United States established that 82% of them used Internet-based resources including Up-To-Date, PubMed, and the Crohn’s and Colitis Foundation of America (CCFA) websites to access update information on IBD management and patient health quality of life [40].

Indisputably, to date, both gastroenterology trainees and specialists actively use online resources to meet their educational curiosity and clinical practice needs in IBD management settings. It is known that these online resources are likely as effective as conventional methods of instruction [41]. In this literature review article, we describe existing web based IBD e-learning resources for all kinds of physicians and patients. Our goal is to provide a guide to physicians, especially in developing nations, where there is a rapid increase of IBD incidence and prevalence, but less learning resources, to gain and maintain state-of-the-art knowledge and up to date skills in IBD management. Some of these resources also have interactive components that allow learners to communicate with their peers and experts in IBD worldwide [38].

## 4. Literature Search Methods

We performed a literature search according to PRISMA-P [42]. We conducted and managed literature searches in PubMed to pinpoint peer-reviewed articles associated to web-based educational information material for IBD. We also used other search engines including Start page (www.startpage.com), Google.ca, Medical Literature Analysis and Retrieval System Online (MEDLINE), the Excerpta Medica database (EMBASE), Current Nursing and Allied Health Literature (CINAHL), the Cochrane library, Google^®^ and Web of Science to find websites entailing educational information material about IBD. Search results were effectuated using the following search maneuver: “IBD” or “UC” or “CD” and “online” and “resources” or “CME” or “educational”. In addition, we carried out targeted searches by browsing websites of national or international foundations/organizations or societies related to IBD.

We also performed manual searching to assess and examine the contents of each web site. We did not use any specific medical website tool metrics to assess the quality of the educational web sites. Journal articles from our PubMed search that had been published in the last 10 years were considered for review. Website inclusion criteria are as follows: active websites in English that are related to IBD education, including those that provide CME courses targeting patients and undergraduate medical students as well as postgraduate medical education and healthcare professionals.

We downloaded or manually entered references from all sources into the online Endnote reference manager (http://www.myendnoteweb.com) to record each website’s name/organization, year, access date, and the URL.

### 4.1. Websites of International and National Societies

#### 4.1.1. European Crohn’s and Colitis Organization (ECCO):

The ECCO has supported and contributed substantially to the education of GI experts in IBD [43]. The ECCO website provides free access to preceding published ECCO Guidelines and the ECCO e-Guide, a toolkit accommodating a collection of algorithms based on the ECCO guidelines, GI disorder disease information, disease undertaking/activity calculators, and other advantageous resources [44].

In 2013, ECCO begun e-CCO [45], an online learning podium aimed at improving overall IBD patient care by contributing a comprehensive educational amalgamation for healthcare professionals. The e-CCO platform is divided into IBD basics, eLibrary, e-Courses, Advanced Topics, and the ECCO IBD Curriculum, all of which require membership for access. The e-library consists of abstracts, presentations (videos and/or slides), and webcasts from the ECCO congresses, created and delivered by gastroenterologist experts in IBD. The current e-CCO learning portfolio contains as many as 24 extensive e-Courses based on the ECCO guidelines, and over 40 original videos and podcasts focused on basic topics on IBD and current controversies in disease management. The e-courses are accredited, and participants who pass the test at the end of the courses will receive a certificate. The participants will also receive feedback during the courses and after the test.

The ECCO IBD Curriculum is a path-breaking framework for ECCO’s educational program activities and engages as its fundamental educational core. It serves as a guide for healthcare providers interested in IBD, the index of the entire on-line ECCO content, and an educational tool for physicians. The ECCO IBD Curriculum is organized into 16 broad topics extending from IBD and treatment to the management of IBD patients. Each domain within the curriculum is continually enriched and updated with novel new material from the ECCO educational and scientific update activities. The purpose of the ECCO IBD Curriculum mission is to provide gastroenterologists with the knowledge and skills necessary for them to become experts in IBD.

#### 4.1.2. Crohn’s and Colitis Foundation of America (CCFA):

The Virtual Practical Preceptorship Program. The CCFA website offers information about IBD for physicians and their patients [46]. CCFA also offers the Virtual Preceptorship program [46]. The Programs and Materials displayed on its website improve physician training in the diagnosis and treatment of IBD using five online reciprocal and accredited enterprises. Further, in the same section, there are also free educational brochures and specific spreadsheets that furnish current information on IBD and treatment course of actions.

#### 4.1.3. Rising Educators, Academicians and Clinicians Helping IBD (REACH-IBD):

REACH-IBD was founded under the patronage and guidance of CCFA. Its objective is to meet and disseminate the educational needs of eligible trainees and all faculty members interested in IBD. It supports mentorship by experienced experts, fosters collaborative research among earlier investigators, provides guidance on career development and trajectory, and obtains best practices for patient management through educational and career enhancement seminars, mentoring initiatives, networking programs, research partnerships, enterprises within CCFA, and trainee educational components [47].

In August 2016, REACH-IBD and the University of Nebraska Medical Center cooperatively launched the IBD Clinical Practice Video Series [48], a year-long accredited online series. Using videos and quizzes consisting of the most recent information, this program addresses knowledge gaps among trainees by covering four topics on IBD treatment and the management of specific IBD-related situations including postoperative subsequent recurrence of Crohn’s disease, pouch endoscopy, pregnant patients with IBD, complications related to IBD, and advanced treatment approaches. The modules are free, exceedingly educational and accessible and include a pre-presentation quiz. Unfortunately, this training program no longer provides pre- and post-learning assessments/evaluations, and educational credit.

#### 4.1.4. Canadian Association of Gastroenterology (CAG):

The CAG has successfully developed and launched ePortal in the Education section of the CAG website [49] ePortal is an accredited initiative devoted to updating the knowledge of CAG members on various topics in digestive system diseases and contributing to their Maintenance of Certification (MOC) requirements. ePortal contains assembled presentations and videos related to GI practice primarily from previous national [Canadian Digestive Disease Week (CDDW)] or local meetings. The site is coordinated into ePortal course categories, and the IBD branch involves 97 courses indexed in chronological order rather than by subject. ePortal instinctively saves the history of each member’s educational program enterprises, which can be reviewed and printed at any time for free.

#### 4.1.5. The American Society of Colon and Rectal Surgeons (ASCRS): Online Learning Center (OLC).

The aim of the ASCRS-OLC is to centralize important educational resources so that its members can easily access and select what they need. ASCRS-OLC offers a solid foundation in the IBD evaluation and management. This site, http://education.fascrs.org/, features multiple online educational activities, including the Colorectal Educational Systems Template (CREST) and the Colon and Rectal Surgery Educational Program (CARSEP).

**CREST** is an online learning program that offers CME credit. It features modules categorized by disease or topic. Learning modules are created using both original content and content from The ASCRS Textbook of Colon and Rectal Surgery. The modules consist of narrated PowerPoint presentations and core subject presentations from ASCRS Annual Meetings as well as hundreds of radiological, endoscopic and anatomical images.

**CARSEP** consists of nearly 250 questions categorized by the six pillars of colorectal surgery, followed by a 100-question self-assessment exam. Optimized for use on a desktop, smartphone, or tablet, CARSEP IX may be used to prepare for the American Board of Colon and Rectal Surgery (ABCRS) examination or to stay current with the latest practice recommendations. CARSEP offers 50 CME credits upon successful completion of the self-assessment exam that can be applied towards Part 2 of the ABCRS MOC program.

##### ASCRS International Travel Scholarship Criteria:

This section provides funding for junior colorectal surgeons residing outside Canada and the United States, to give support with travel to the U.S. for the educational encounter of participating the Annual Scientific Meeting of the ASCRS. Apparent ASCRS International Traveling Scholarship Awardees from African countries (Dr. Ayasiga Herman, Tanzania, Dr. Alex Elobu, Uganda and, Dr. Olusegun Alatise, Nigeria attended ASCRS annual meetings in 2017, 2015 and 2013, respectively. They were in conformity of being uninformed about IBD in their respective countries. In fact, they did not know the disease clinical presentations. Dr. Zaheer Mooll, a 2017-awadee from South Africa was informed but was not proficient about IBD. These young surgeons are suggesting that a research component be introduced into the scholarship in some way. Furthermore, if the number of awardees from developing nations could increase for more young colorectal surgeons to be exposed to excellence.

#### 4.1.6. The American Gastroenterological Association (AGA):

The American Gastroenterological Association (AGA) is the committed platform of the GI community spotlighting IBD education to help healthcare providers and patients suffering from IBD. AGA publishes guidelines and education materials, and ensures clinicians have a voice when it comes to policies that affect them and their patients [https://apps.apple.com/us/developer/american-gastroenterologicalassociation/id472976635].

### 4.2. University Websites

#### 4.2.1. “IBD LIVE” Webcast Program:

In 2009, University of Pittsburgh launched the “IBD LIVE” webcast program [50, 51] an interinstitutional/ interdisciplinary video conference that allows remote participation in live IBD case discussions on Thursday early mornings from 7:00 AM to 8:00 AM EST. Each conference session covers two IBD cases. Presently, over 25 academic IBD centers on the east coast of the United States participate in this conference, where attendees can interact with experts in IBD in an active learning setting. Enrollment and access to the program is complimentary via an easy online registration process. The webcast associates can follow and view the discussion and submit query or comments and suggestions via a chat feature mechanism. Previous webcasts are archived and accessible to all participants. IBD LIVE is accredited by the UPMC Center for CME in the Health Sciences (CCEHS).

This program is a compelling educational resourcefulness that enables remote participation in a CME-approved interdisciplinary conference. IBD LIVE improves the participants’ knowledge. It also allows them to exchange viewpoints and ideas with other colleagues and experts in IBD, collaborate with peers from other centers, and ultimately improve their ability to care for IBD patients.

#### 4.2.2. IBD Groups Websites

##### The IBD Working Group (IBDWG):

The IBDWG conducts an educational symposium/ forum for healthcare professionals specialized in IBD and aims to promote and improve the quality of IBD patient care [52]. The website contains immense-quality and clinically aligned educational supports focused on IBD prepared in partnership with top experts specialized in IBD from the United States, Canada and Europe. The content of the site is available via complimentary subscription and is organized into sections where presentations/ deliveries (slides with/without audio) and other educational material are listed. While post-activity tests with feedback are available, the website stopped awarding CME credit in 2016 and the content has not been updated.

##### IBD dialogues and E- mentoring in IBD:

Mentoring in inflammatory bowel disease (MIIBD) is an innovative and successful annual national conversion for Canadian GI experts (The Master Class) that takes place in Toronto, Canada [53]. It also operates regional satellite meetings, a website, a newsletter series, and regular electronic communications that answer key clinical queries with new information generated from research conducted by Canadian and international experts in IBD.

Launched in 2004, IBD Dialogue is a quarterly electronic newsletter based on subjects/ topics presented at the annual Mentoring in IBD: The Master Class symposium [54] IBD Dialogue reports on new developments and best practices in IBD management as well as case discussions involving experts in IBD.

Launched in 2008, E-mentoring in IBD is a reciprocal scientific e-bulletin on latest issues in IBD management advances. It is published twice a month and delivered to its subscribers via email [55]. E-Mentoring in IBD examines clinically relevant questions reported in current publications and provides two-sentence conclusions based on the reported findings. A measurement of the difference of evidence and hyperlinks to the sources are also provided.

The Mentoring in IBD website is complimentary to access. Users can find accredited educational materials, browse or download publications including previous IBD Dialogue and E-mentoring newsletters, and watch videos conferred by experts in IBD. Subscription to the newsletters and bulletins is complimentary [53].

### 4.3. Industry-Sponsored Websites

#### 4.3.1. Criterion for Standardizing the Endoscopic Evaluation of Mucosal Lesions in IBD (SEEMLI):

SEEMLI is a CAG-accredited curriculum supported by AbbVie. Its objective is to enhance the proficiency of gastroenterologists in performing endoscopies as well as to improve their practice experience and skills in using various endoscopic scoring approaches [56]. The scheme focuses on the most common endoscopic scoring methods used in clinical practice: the Simple Endoscopic Score for CD (SES-CD), the UC Endoscopic Index of Severity (UCEIS), the Mayo Endoscopic Score (MES) for UC, and the Rutgeerts score for post-ileocolic resection of CD.

The initiative program provides important information on how to engage each of these practices, discusses the pros and cons of each approach, and furnishes practice opportunities using endoscopic videos. Subscription is complementary, and users can access their dashboards to view the courses they have taken and their respective progress.

#### 4.3.2. IBD Talks and IBD Points:

IBD Talks and IBD Points were co-initiated by the CAG and AbbVie through an educational grant [57]. These online modules are educational initiatives refined to contest the learning requirements of practicing GI experts in IBD on how motivational communication could be utilized in clinical setting. They were developed by a group of interdisciplinary faculty members that includes gastroenterologists, nurses specialized in IBD, and a motivational communication expert.

These modules are accredited self-assessment programs as defined by the Maintenance of Certification Program of the Royal College of Physicians & Surgeons of Canada, approved by CAG on March 2016. The accreditation has been renewed in March 2019. Each module is worth two credits and consists of a pre-test, learning module, post-test, self-assessment evaluation, and program evaluation. Subscription to users can access their dashboards to view their respective progress for each module.

### 4.4. Independent Resources

#### 4.4.1. Imedex E-Learning Center:

Imedex^®^ is an industry leader in accommodating certified, independent CME for healthcare professionals. It conducts immense-quality scientific enterprises in multiple specialties that aim to advance disease treatment and patient care to improve quality of life. The e-learning material includes video and audio recordings of interviews, debates, and panel discussions involving world-renowned experts that present clinically relevant information resulting from the latest research in various areas in medicine, including gastroenterology and IBD [58]. Subscription is complementary, and the activities are accredited and put together into lists for each segment of GI specialty. Subscribers can sign up and request for email updates and alerts to all types of educational materials.

#### 4.4.2. You and IBD:

This website is executed to safe guide and update IBD patients, their families, and their caregivers on the latest available information advances on IBD [59]. Upon free registering, users can access and download complementary educational materials as well as receive updates and notifications from the website with excellent portrayed animations, patient slideshows, a self-evaluation quiz, a feedback survey, and a boundless library of visual tools. Additional topics presented range from causes of IBD and the diagnosis of CD or UC to those related to lifestyle choices, diet, medications, and surgeries used to manage and treat IBD.

#### 4.4.3. MyCME:

Haymarket Medical Education (HME), a medical education team, has initiated MyCME to offer autonomous continuing education curriculum to clinicians, clinician assistants, pharmacists, nurses, nurse practitioners and other healthcare professionals [60]. With a complimentary subscription to the MyCME website, users can freely access CME activities listed by specialties. To receive a certificate, candidates must read the learning missions and disclosure statements, complete a pre-test, study or watch the educational curriculum plan, and fulfil the post-test and program activity evaluation form. The online certificate can be saved in the user’s Profile/CME History on the website and can be accessed and downloaded at any convenient time. The MyCME application component is also available as complimentary to download.

#### 4.4.4. CME Outfitters (CMEO):

CMEO is a sovereign support of accredited, evidence-based medical educational enterprise. Operational since 2002, it aims to advance patient care by enhancing clinical proficiency of healthcare professionals including clinicians, clinician assistants, psychologists, nurses, nurse practitioners, pharmacists, social workers, clinical case managers, and other healthcare providers [61]. On the CMEO website, users can find CME activities organized by date, topic (for example, Gastroenterology: IBD, CD, or UC, etc.), credit type and specialty. Materials are available and are presented in multiple formats designed to satisfy multiform learning choices, encompassing internet webcasts (live and archived), nationally televised satellite broadcasts (live and recorded), journal club webcasts, chart review webcasts, major medical meetings, podcasts, symposia, and conferences. Each CMEO activity involves a knowledge evaluation during the credit request process that includes a pre-test, a scored post-test accompanied by performance feedback, and an evaluation of the activity. Subscription is complementary.

#### 4.4.5. GastroCE:

GastroCE is another self-reliant grasp of accredited evidence-based medical educational program that provides articles, videos, case report studies, lectures, and CME modules wrapping different areas in IBD [62]. Each CME activity involves a pretest knowledge evaluation and a scored post-test. Individual self-evaluation tests are also available. The history of participation for each user is stored in the user account. Resources for patients are retained available on this website. Subscription is complementary.

#### 4.4.6. Medscape:

Medscape is a well-established global website for clinicians and other healthcare professionals. Subscription is complementary. Users can access the latest researched medical update and expert viewpoints, indispensable drug and disease update information, and relevant professional educational enterprises including accredited CME activities. Medscape also offers the Medscape, MedPulse News and the CME and Education applications.

In the Medscape Gastroenterology section, users can find the up-to-date news about different digestive system disorders including IBD [63]. The Inflammatory Bowel Disease CME Learning Center is available under the CME & Education List and provides current accredited CME exertions [64].

### 4.5. Other Websites

There are many other websites that provide educational material for IBD which we are unable to cover in this review. Some examples include IBD: Vital Concepts and Management Paradigms on the American College of Physicians (ACP) website [65], the Cleveland Clinic Center (CCC) for continuing education website [66], MedPage Today [67], and the Inflammatory Bowel Disease Toolkit [68].

More accredited educational material related to IBD are available on the American College of Gastroenterology (ACG; ACG Education Universe) [69] and the American Gastroenterological Association (AGA; GI self-assessment modules SAM, Digestive Diseases Self-Education Program, DDSEP) [70] websites. This material covers various topics on GI disorders including IBD. In addition, AGA’s clinical guidelines endorses guidelines that meet the National Academy of Medicine’s rigorous criteria with evidence-based recommendations to assist guide clinical practice decisions based on rigorous systemic reviews of the medical literature. Further, AGA runs an IBD parenthood project which provides information to women with IBD and how can have healthy babies [71]. The IBD Parenthood project further helps gastroenterologists provide care during all stages of family planning such as: groundbreaking medical research in IBD - a family affair, supporting IBD patients in family planning and pregnancy, how to care for IBD patients in pregnancy and, what is the IBD in Pregnancy Clinical Care Pathway? Eventually, subscribers of the AMEDEO Medical Literature Guide get leaflet and/or newsletters that include overviews of new articles published on pre-selected topics (for example, IBD) and substantiated journal subsets [72]. Comparably, PubMed sends subscribers regular email messages with new search results based on prior saved search terms [73].

Subscription to both resources is free.

## 5. Summary

This summarized review article may reflect differently between demographic populations of the developing nations, especially African communities. However, it recommends emerging trends given the significantly loitered presentations and stresses the significance of its awareness locally. This event gives a foundation for further community and tertiary-based-exploration into the pathogenesis of IBD as its accruals significance in developing nations, especially African nations [74].

The emergence of new treatments, the shift of therapy goals from controlling symptoms to endoscopic mucosal healing in the treat-to-target approach, overtures in imaging technology and interventional surgical techniques, and the adjustment to a more patient-centered perspective in IBD management and patient care have made IBD management very difficult, demanding and challenging. Healthcare professionals treating IBD patients need to navigate these challenges by founding, reinforcing, and managing a strong core of knowledge and skills related to IBD management. Moreover, it has become more common for patients to use the Internet to access information about their health concerns. Therefore, healthcare professionals should be well-versed in the latest information regarding IBD to address their patients’ questions and concerns.

Traditionally, healthcare professionals would acquire new knowledge through supplemental reading of compatible textbook or journals, attendance of congresses and meetings, and group discussions. Recently, technology-enhanced learning using the Internet as its major source of educational material increases the options for information and knowledge acquirement. This new way of learning may be more time- and cost-efficient than conventional methods.

In this review article, we explore online e-learning resources for IBD. There are many diverse resources that provide supportive information material about new pharmaceuticals or strategies in order to advance or maintain the knowledge of healthcare professionals as well as enhance their experience and management skills. We describe some examples of on-line-sites that offer considerable and different educational material associated to IBD (Table 1). Interested healthcare professionals should visit and navigate the admissible websites that cater to their educational needs. It would also advisable for them to get familiar or affiliate with a few certified Internet resources that they can recommend to their patients. Examples of these resources include the ECCO IBD Curriculum [13], CMEO [29], the GastroCE [62], the IBD LIVE webcast program [19], You and IBD [17], and the E- mentoring in IBD websites [23]. These resources provide some of the most impressive, novel, and well documented educational activities related to IBD and IBD advances.

## 6. Crystals

With the emergence of new internet communications technology, Web-based resources can adequately address the educational requirements of both IBD patients and healthcare professionals who deal with IBD. These resources can also subsidize to the advancement of IBD care, patient management, and patient quality of life outcomes. It is to the advantage of IBD related issues that future studies should investigate the quality and the utility of these websites to find ways to regularly, as needed, improve them.

## 7. Conclusion

In this century, western culture and urbanization represent a major demographic shift in developing and African nations. In western nations IBD rates are beginning to level off but there is increasing incidence and prevalence in developing countries possibly because these nations are transitioning to more westernized, industrial and urbanized population societies. This implies there may be an environmental etiopathogesis trigger(s) for the disease, as the onset is too rapid to be accounted for by genetic changes. Knowledge about the diagnostics of IBD is tantamount to the preparation of clinical infrastructure, resources, and personnel to manage IBD.

### 7.1. Lessons for Practice

In most developing nation’s educational curriculum, the locally graduated healthcare professionals are not trained about IBD and have therefore limited or no knowledge about the disease. In these countries IBD is often misdiagnosed as chronic diarrhea and treated as bacterial and/or parasitic infectious dysenteric disease (e.g. shigellosis and/or amoebiasis, etc.). The healthcare systems, both at the primary care and referral levels, often do not have the ordinary clinical supervision and laboratory assessments required for monitoring patients. With the emergence of advanced communications technology, the internet offers diverse, substantial, easily accessible, and up to date IBD-related educational resources that are more time- and cost-effective to assist physicians and patients worldwide. Through this approach the developing nation’s educational institutions have access and privilege to use these mentioned scientific societies.

## Figures and Tables

**Table 1:** Inflammatory bowel disease educational resources for healthcare professionals and patients

		Website	
	Name	Address	Educational activity type
SOCIETIES			
ECCO	e-CCO	https://e-learning.ecco-ibd.eu/	IBD curriculum, archived videos, webcasts, CME, etc.
CCFA	Virtual Preceptorship	http://www.crohnscolitisfoundation.org/science-and-professionals/programsmaterials/virtual-preceptorship.html	videos, brochures, CME
	REACH-IBD	http://programs.rmei.com/IBDKnowledgegap	videos
CAG	*e*Portal	https://www.cag-acg.org/education/eportal	videos, slide shows, CME, MOC
ACG	ACG Education Universe	http://universe.gi.org	accredited courses, CME, MOC
AGA	AGA Education	http://www.gastro.org/education	accredited courses, CME, MOC
UNIVERSITIES			
University of Pittsburgh	IBD LIVE	https://services.choruscall.com/links/UPMC/ibd/	live webcasts, archived webcasts
IBD GROUPS			
The IBD working group	IBDWG	http://www.ibdwg.org/index.cfm	videos, slide shows
Mentoring in IBD	IBD dialogue	http://www.mentoringinibd.com/category/ibd-dialogue/ classic-edition/	bulletin via email
	*e* mentoring IBD	http://www.mentoringinibd.com/category/e-mentoring/	newsletter via email
INDUSTRY SPONSORED			
	SEEMLI	https://www.seemli.ca/Dashboard-/MyCourses	videos, power point presentations, CME
	IBD Talks & Points	https://www.ibdtalkspoints.ca/login/index.php	videos, CME
INDEPENDENT RESOURCES			
Imedex	Imedex *E*-learning		videos, CME
You and IBD	Center	http://elc.imedex.com/	animations, slide shows, quiz
Haymarket Medical	You and IBD	http://www.youandibd.com/en-ibd/home	videos, CME
Education	MyCME	http://www.mycme.com/	videos, CME
CME outfitters	CME outfitters	https://www.cmeoutfitters.com/	articles, lectures, videos, case
GastroCE	GastroCE	https://cme.healio.com/gastroce/	studies, CME
Medscape	IBD CME Learning Center	https://www.medscape.org/resource/ibd/cme	news, videos, CME
MEDPAGE TODAY	Gastroenterology	https://www.medpagetoday.com/gastroenterology	news, videos
AMEDEO	Literature Guide in IBD	http://amedeo.com/medicine/ibd.htm	journal scan email
PubMed	My NCBI	https://www.ncbi.nlm.nih.gov/sites/myncbi/searches/	journal scan email
